# Evidence for Declining Numbers of Ebola Cases — Montserrado County, Liberia, June–October 2014

**Published:** 2014-11-21

**Authors:** Tolbert G. Nyenswah, Matthew Westercamp, Amanda Ashraf Kamali, Jin Qin, Emily Zielinski-Gutierrez, Fred Amegashie, Mosaka Fallah, Bernadette Gergonne, Roselyn Nugba-Ballah, Gurudev Singh, John M. Aberle-Grasse, Fiona Havers, Joel M. Montgomery, Luke Bawo, Susan A. Wang, Ronald Rosenberg

**Affiliations:** 1 Liberia Ministry of Health and Social Welfare; 2 National Center for Immunization and Respiratory Diseases, CDC; 3 Epidemic Intelligence Service, CDC; 4 Los Angeles County Health Department; 5 National Center for Chronic Disease Prevention and Health Promotion, CDC; 6 Center for Global Health, CDC; 7 Montserrado County Community Health Department; 8 Médecins Sans Frontières; 9 Liberian Red Cross Society; 10 International Federation of Red Cross and Red Crescent Societies; 11 National Center for Emerging and Zoonotic Infectious Diseases, CDC

The epidemic of Ebola virus disease (Ebola) in West Africa that began in March 2014 has caused approximately 13,200 suspected, probable, and confirmed cases, including approximately 6,500 in Liberia (1,2). About 50% of Liberia’s reported cases have been in Montserrado County (population 1.5 million), the most populous county, which contains the capital city, Monrovia. To examine the course of the Ebola epidemic in Montserrado County, data on Ebola treatment unit (ETU) admissions, laboratory testing of patient blood samples, and collection of dead bodies were analyzed. Each of the three data sources indicated consistent declines of 53%–73% following a peak incidence in mid-September. The declines in ETU admissions, percentage of patients with reverse transcription–polymerase chain reaction (RT-PCR) test results positive for Ebola, and dead bodies are the first evidence of reduction in disease after implementation of multiple prevention and response measures. The possible contributions of these interventions to the decline is not yet fully understood or corroborated. A reduction in cases suggests some progress; however, eliminating Ebola transmission is the critical goal and will require greatly intensified efforts for complete, high-quality surveillance to direct and drive the rapid intervention, tracking, and response efforts that remain essential.

## ETU Admission Data

ETU admission data include all admissions to the four Montserrado ETUs* as reported to the Ministry of Health and Social Welfare for the period June 13–October 26, 2014. Of 2,916 patients admitted, admission dates were available for 2,768 (95%). For purposes of this analysis and because ETU admission data do not contain information on symptoms or Ebola risks, classification of cases depended on ETU documentation of negative Ebola RT-PCR test results: 1) a non-Ebola case was defined as a case in which a patient was admitted to an ETU but released as Ebola-negative based on ETU documentation of a negative Ebola test result by RT-PCR and there was absence of any ETU-documented positive Ebola test result, and 2) an Ebola case was defined as a case in which a patient did not have an ETU-documented negative RT-PCR test result, even if laboratory results were not recorded (including confirmed cases [those with ETU documentation of a positive RT-PCR test result], as well as probable and suspected cases for which there were no ETU-documented negative RT-PCR test results). Release as a non-Ebola patient was based on a patient having two consecutive negative Ebola RT-PCR tests at least 72 hours apart. It was not possible to make exclusions based on county of residence.

**FIGURE 1:**
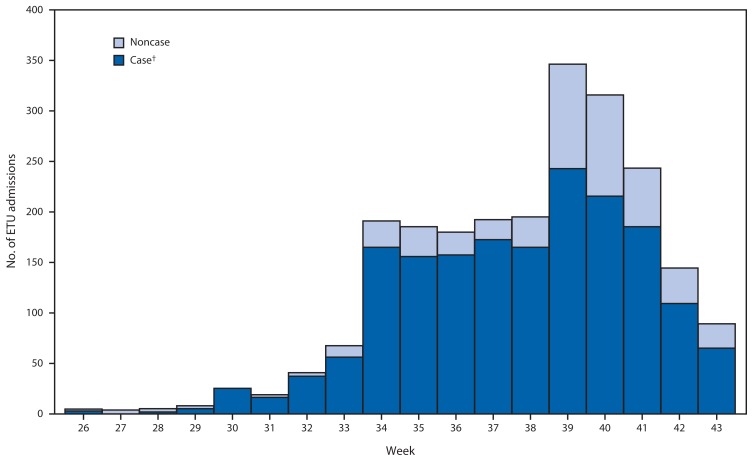
Number of admissions to Ebola treatment units (ETUs),* by week and case status — Montserrado County, Liberia, June 13–October 26, 2014 * ETUs located in Montserrado County are ELWA (Eternal Love Winning Africa)-2, ELWA-3, Island Clinic, and JFK (John F. Kennedy) ETUs. ELWA-2 began operating on July 20, ELWA-3 and JFK began operating on August 17 (week 34), Island Clinic began operating on September 20 (week 38), and JFK closed on October 7 (week 41). ^†^ Includes cases that have been confirmed with ETU documentation of positive Ebola reverse transcription–polymerase chain reaction (RT-PCR) results, as well as those probable and suspect cases for which there are no ETU-documented negative RT-PCR results.

The number of admissions to ETUs rose to a maximum of 255 patients during epidemiologic week 39 (beginning September 22) and then declined by 67% to approximately 70 per week by week 43 (October 26) (Figure 1). Ebola cases and noncases followed this trajectory. The number of beds available for Ebola patients rose substantially, from fewer than 100 to more than 500 beds during the study period, moving from an initial shortage to a surplus. Because patients were turned away from ETUs due to bed shortages during the period when the number of Ebola cases was rising, the number of ETU admissions was effectively capped during various weeks; thus, the number of patients who could have been admitted during August and September is not depicted (Figure 1). Eternal Love Winning Africa (ELWA)-2 ETU began operating on July 20, ELWA-3 and John F. Kennedy (JFK) ETUs began operating August 17, Island Clinic ETU opened on September 20, and JFK ETU closed October 7. Trends in ETU admissions are affected by changes in the number of available ETU beds and might be influenced by changes in migration of patients to and from other counties. However, availability of ETU beds was stable or increasing during the last 4 weeks analyzed, when there were also no large shifts in patient migration to account for the decline of ETU admissions observed.

## Ebola Laboratory Test Data

Laboratory test results for Ebola by RT-PCR were provided by three dedicated laboratories processing samples for Montserrado County for the period August 18–October 26, 2014.† Results were available for 5,866 specimens, representing 4,077 patients. Because patient-level identifiers were not assigned consistently, a unique identifier was created using a patient’s initials, sex, age, and home location to link multiple specimen records for the same patient. In total, 405 specimens (7%) lacked the information necessary for assignment of an identifier and were excluded. Test week was based on when the first specimen was taken. Results from patients with reported home locations outside Montserrado County were excluded.

**FIGURE 2:**
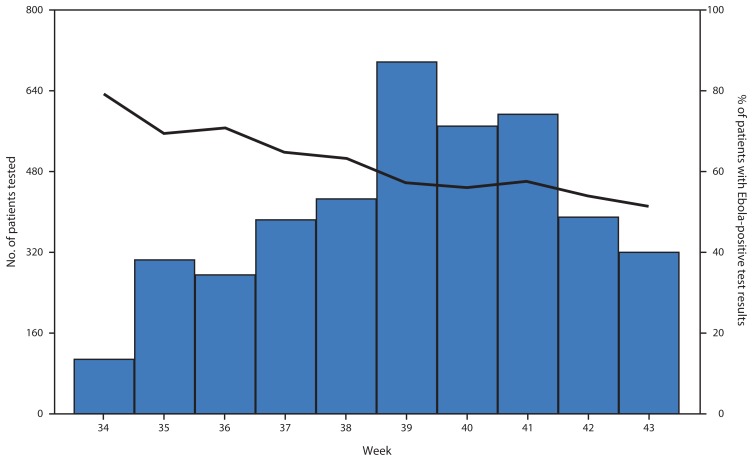
Number of patients with RT-PCR tests performed (bars) and percentage of patients with Ebola-positive test results (line), by week — Montserrado County, Liberia, August 18–October 26, 2014* **Abbreviation:** RT-PCR = reverse transcription–polymerase chain reaction. * Laboratory results are calculated per patient by week of first positive test performed. Repeat tests for a given individual were removed. CDC/National Institutes of Health and Island Clinic laboratories began operating on August 20 (week 34) and October 2 (week 40), respectively. The Liberian Institute of Biomedical Research laboratory began testing specimens on August 7 (week 32); however, data from before week 34 were excluded because of the lack of sufficient information to identify multiple samples from individual patients.

Mirroring ETU admissions, the number of patients tested increased through week 39, reaching an average of 100 patients per day, followed by a decline through week 43, at an average of 46 patients per day. The percentage of patients with Ebola-positive RT-PCR test results, excluding repeat tests for individual patients based on the unique identifier, declined gradually over the entire period, from a maximum of 79% positive at week 34 (August 18) to 51% positive by week 43 (October 20) (Figure 2).

## Body Collection Data

**FIGURE 3:**
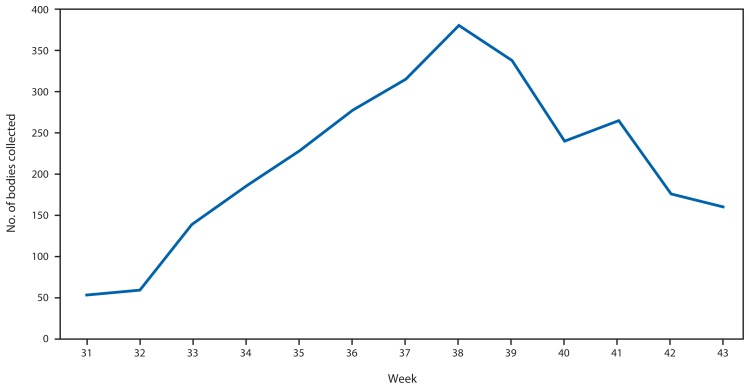
Number of bodies collected by IFRC and ELWA-3, by week — Montserrado County, Liberia, July 28–October 26, 2014 **Abbreviations:** IFRC = International Federation of Red Cross and Red Crescent Societies; ELWA = Eternal Love Winning Africa.

Since late July 2014, the International Federation of Red Cross and Red Crescent Societies (IFRC) has been responsible for the collection and cremation of all dead bodies from ETUs (except ELWA-3) and bodies from the community. For the period July 28–October 26, a total of 2,234 bodies were collected by the IFRC. The majority (1,179 [53%]) were collected from homes or other community settings, 744 (33%) from ETUs, 194 (9%) from non-ETU health facilities, and 117 (5%) from unknown locations. ELWA-3 operates its own crematorium. To examine the trend of the total number of bodies collected, ELWA-3 records (n = 578) were combined with the totals from IFRC (Figure 3). The number of bodies believed to be the result of an Ebola-related death rose to a maximum in week 38 (September 15), with 380 bodies collected, and then declined to 160 by week 43 (October 20). The pattern was similar for both the IFRC and ELWA-3.

### Discussion

The decline in the number of Ebola cases in Montserrado County from a peak in mid-September was indicated by three data sources: ETU admissions (73% decline), laboratory results (58% decrease in Ebola-positive test results), and body collection (53% decline). The patterns of change in the three indicators were similar, and there is no apparent common source of systematic error that can account for simultaneous decline in all three indicators. These analyses support accumulating anecdotal evidence that cases in the county were substantially lower in late October than 2 months earlier.

This analysis depends on existing program records collected by several independent groups. The magnitude of the epidemic overwhelmed the routine data collection system, and each of the data sets analyzed contained incomplete or indecipherable records that were removed before analysis. The manual linkage of patient records required substantial data processing and limited the ability to compare information between data sources. Assignment of a unique patient/case identifier at first patient contact would improve surveillance and data management.

The completeness of records was further compromised by refusal of an unknown number of persons to report cases or burials (Montserrado County Contact Tracing Team, personal communication, 2014). The need to cremate Ebola-related dead bodies has encountered resistance from the local population, raising the possibility that bodies might have been hidden and independently buried. A rapid community assessment performed in October examining community perceptions and avoidance of cremation, however, suggests no increase in frequency of such secret burials during September and October to account for the recorded decrease in body collection (African Union and CDC, unpublished data, 2014).

The numbers of cases projected, based on an exponential growth model that used early epidemic trends and assumed no effective interventions, did not materialize (3,4). However, Ebola is far from eliminated in Montserrado, and the continued weekly discovery of new cases indicates that elimination from the county could be lengthy and progress could be reversed. The medical and humanitarian response to Ebola in Liberia, including isolation and care of patients, contact tracing and management, supportive care, safe burials, and the establishment of a coordinating incident management system, have been augmented by an intense and pervasive program by government and partners to educate the public on symptoms, prevention, and care of the infected, as well as the appropriate handling of the bodies of Ebola victims (5,6). Community action, aided by use of this information, might have substantially contributed to the decline. Most Liberian communities, whether villages or dense urban blocks, have respected, informed leaders who have in many cases initiated protective actions, such as contact identification, active case detection, insistence on safe burials, and isolation or quarantine measures, usually in the aftermath of a neighbor’s infection. Enlistment of community leaders as key informants to develop a comprehensive surveillance network will be an essential component of ongoing surveillance and response. Equally critical is the need to intervene rapidly with isolation of and care for the sick, as well as rapid removal of the bodies of Ebola victims for a respectful but safe burial. This is especially important if the epidemic matures into a widespread patchwork of small outbreaks that threaten to expand in the absence of quick and decisive responses.

What is already known on this topic?

The epidemic of Ebola virus disease (Ebola) began as small foci of cases in the border regions of Guinea, Sierra Leone, and Liberia before March 2014. It has now infected approximately 13,200 persons in eight countries. Liberia has had the most reported cases, approximately half of which have occurred in Montserrado County.

What is added by this report?

A decline in the number of Ebola cases in Montserrado County from a peak in mid-September is indicated by three data sources: admissions to Ebola treatment units (73% decline), laboratory results (58% decrease in patients with Ebola-positive test results), and body collection (53% decline).

What are the implications for public health practice?

Decreases in Ebola in one county indicate the potential for and challenge of elimination of Ebola. There remains the risk that progress can be reversed as long as new cases continue to be identified. Rapid response teams, effective contact tracing, prompt isolation and care, infection control throughout the health care system, and increased emphasis on working with networks of community leaders to report and respond to cases will be critical to eliminating human-to-human transmission.

## Footnotes

* ETUs located in Montserrado are ELWA-2 (Eternal Love Winning Africa), ELWA-3, Island Clinic, and JFK (John F. Kennedy) ETUs.

† Laboratories located in Montserrado County and included in this analysis included 1) the CDC/National Institutes of Health (NIH) laboratory, which tested specimens from ELWA-2 and ELWA-3; 2) the Island Clinic Laboratory managed by the U.S. Navy, which tested specimens for the Island Clinic ETU; and 3) the Liberian Institute of Biomedical Research laboratory operated by NIH and the U.S. Army Medical Research Institute of Infectious Diseases, which received specimens from various other sources.
